# On-Surface Locomotion of Particle Based Microrobots Using Magnetically Induced Oscillation

**DOI:** 10.3390/mi8020046

**Published:** 2017-02-04

**Authors:** U Kei Cheang, Jamel Ali, Hoyeon Kim, Louis Rogowski, Min Jun Kim

**Affiliations:** 1 Department of Mechanical Engineering & Mechanics, Drexel University, Philadelphia, PA 19104, USA; ukc23@drexel.edu (U.K.C.); jna35@drexel.edu (J.A.); 2 Department of Mechanical Engineering, Southern Methodist University, Dallas, TX 75275, USA; hoyeonk@mail.smu.edu (H.K.); lwr24@drexel.edu (L.R.)

**Keywords:** microrobotics, magnetic control, low Reynolds number

## Abstract

The low Reynolds number condition presents a fundamental constraint on designing locomotive mechanisms for microscale robots. We report on the use of an oscillating magnetic field to induce on-surface translational motion of particle based microrobots. The particle based microrobots consist of microparticles, connected in a chain-like manner using magnetic self-assembly, where the non-rigid connections between the particles provide structural flexibility for the microrobots. Following the scallop theorem, the oscillation of flexible bodies can lead to locomotion at low Reynolds numbers, similar to the beating motion of sperm flagella. We characterized the velocity profiles of the microrobots by measuring their velocities at various oscillating frequencies. We also demonstrated the directional steering capabilities of the microrobots. This work will provide insights into the use of oscillation as a viable mode of locomotion for particle based microrobots near a surface.

## 1. Introduction

The concept of using microrobotics for both in vitro and in vivo applications had been well explored; such as micromanipulation and microfabrication [1,2], drug delivery [3,4], tissue manipulation [2,5], and in situ sensing [6], such as in vivo diagnostics [7,8,9]. At the center of the development of microrobotics has been the study of locomotive mechanisms at low Reynolds numbers. The understanding of locomotion in the context of low Reynolds numbers is critical, as the negligible inertia at low Reynolds numbers requires the use of nonreciprocal motion for movement. Because of this, designing locomotive mechanisms has been one of the difficulties in low Reynolds number motion since many conventional swimming strategies, such as a rigid flapping fin, are ineffective at the micro- and nanoscale due to their reciprocity. According to Purcell, the flexible oar is one of the two major classifications of biological mechanisms for low Reynolds number locomotion; the other being the rotating corkscrew [10]. The bending of an oscillating flexible oar allows for nonreciprocal motion; the oscillating flagella of sperm and the beating cilia of protozoa are biological examples of the flexible oar.

To overcome low Reynolds number constraints, various mechanisms and strategies have been employed in a number of different microrobots such as the helical chiral swimmers [11,12,13,14,15,16,17,18,19], the bacteria powered microrobots (BPMs) [20,21,22], the magnetically steered swimming cells [23], the magnetically controlled Mag-μBot [24], the optically-deformed three-bead systems [25], the self-assembled nanoparticle swimmers [26], the biflagellate micro-objects [27], and the chemically-driven robots [28,29,30,31,32,33,34,35]. In this work, we focus on a class of microrobots that utilize their flexible bodies for motion. Notable examples of existing microrobots in this category include microscopic artificial swimmers with flexible DNA linkages [36], flexible nanowire motors [37], MagnetoSperm [38], sperm-shaped microrobots [39,40], and soft micromachines [41].

The chain-like conjugation of beads that makes up a particle based microrobot is flexible since the connections between the beads are not completely rigid. Note that although the previously reported three-bead achiral microswimmers were claimed to be rigid while rotating [42], this does not apply to particle based microrobots undergoing oscillation. A flexible object rotating at a constant rate will eventually reach a steady state shape where the structure of this object can be modeled as near rigid; this was reported for bacterial flagella where a flexible flagellar filament can be modeled as a rigid helix when rotating [43,44,45,46]. This is caused by the constant viscous force experienced by the object under a constant rate rotation at steady state. Viscous forces on an object under oscillation, however, are time varying; thus, the object will undergo deformation in a cyclic manner as seen in the flexible oar example [10].

In this study, we investigate the motion of particle based microrobots induced by an oscillating magnetic field as an alternative strategy of locomotion for near surface movements. The particle based microrobot converts rotational motion into translation motion and swims in the bulk fluid [42,47,48]. When near a boundary, the microrobot’s flow field interacts with the boundary, leading to rolling motion [42]. The rolling motion leads to movement in the direction perpendicular to the swimming direction. Thus, rotation based microrobots, including particle based microrobots and helical swimmers, move diagonally when near a boundary. Oscillation, on the other hand, does not lead to rolling motion. In a situation where a rotating microrobot navigates through tight boundaries, rolling will cause the microrobots to deviate from the expected path. While oscillation based locomotion does not present an advantage in terms of speed, the elimination of rolling can provide reliability in predicting the trajectory for near boundary navigation. Therefore, oscillation can be considered as an alternative locomotive strategy for the particle based microrobots when moving near a boundary.

## 2. Materials and Methods

### 2.1. Fabrication

**Figure 1:**
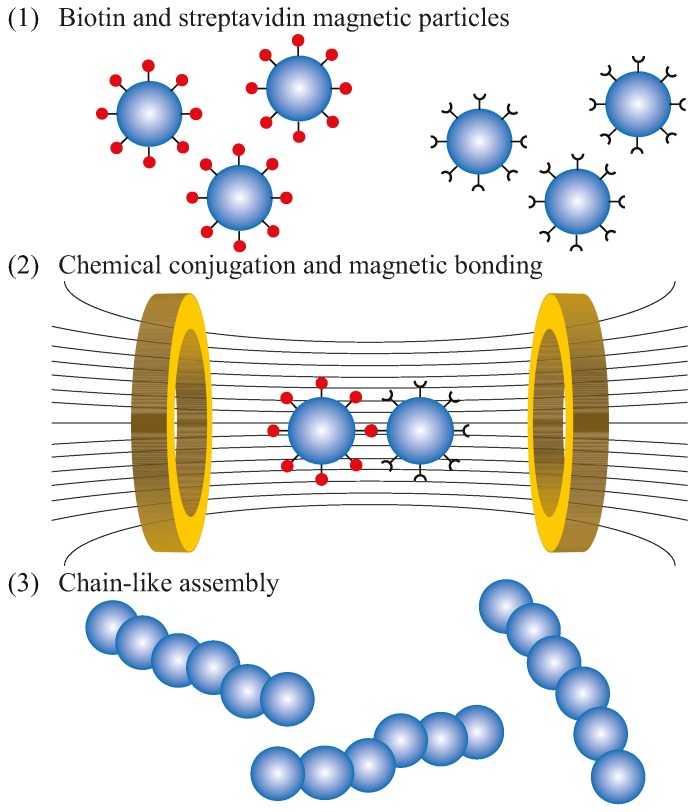
Fabrication process of the particle based microrobots. The process follows three main steps: (**1**) preparation of the biotin and streptavidin coating of the magnetic particles; (**2**) chemical conjugation and magnetic bonding of the coated particles; (**3**) self-assembly of particles into chain-like structures.

The particle based microrobots used in this study consisted of six or more magnetic microparticles (4.35 µm diameter) connected in a chain-like manner. The process of connecting beads using streptavidin-biotin linkages and magnetic attraction is illustrated in Figure 1. First, two batches of coated magnetic microparticles are prepared; one batch with streptavidin coating and the other with biotin coating. The coated particles are commercially available (Spherobeads). Alternatively, particles can be coated using streptavidin and biotin solutions with the same results [42]. Next, the two batches are mixed together and then placed inside a magnetic field. Finally, the magnetized particles will actively move towards each other and then bond together by magnetic forces and the streptavidin-biotin linkages. One of the advantage of using a magnetic field in the fabrication process is the alignment of dipoles, which ensures that the particles bond in a chain-like manner. Note that the chains are not perfectly linearly due to other factors in the mixing process, such as electrostatic forces, surface roughness, sphericity of particles, etc. The connections between the particles in the chain provide flexibility which allows for swimming or fluid transport at low Reynolds number. Previous work on tethered flexible magnetic bead cilia, fabricated in a similar manner as the particle based microrobots, had demonstrated that particle chains can be used to transport fluids at low Reynolds number by using nonreciprocal beating [49,50,51,52]. Similarly, the particle based microrobots are untethered swimmers that can generate a propulsive force when their body oscillate.

### 2.2. Motion Control Using Magnetic Fields

**Figure 2:**
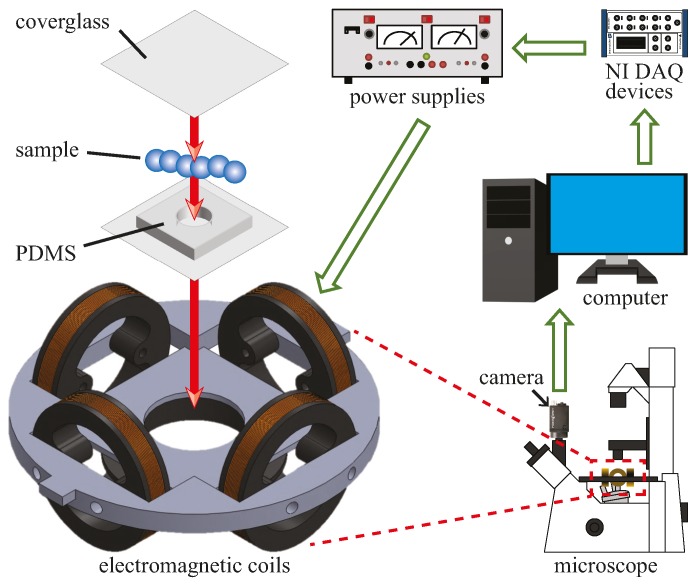
Experiment setup and control system for motion control using oscillating magnetic fields.

There are several advantages in using magnetic fields as the actuation method for microrobots. Magnetic fields can permeate and transmit power over long ranges for propulsion, motion control, and magnetic resonance targeting (MRT) [53,54]. The control system used here was designed to generate an oscillating magnetic field at any desired oscillating frequency and field strength. The system consists of an electromagnetic coil system, three bipolar power supplies (Kepco), a National Instrument (NI) data acquisition (DAQ) device, a computer, an inverted microscope (Leica DM IRB), and a camera (Point Grey). In experiments, we can control the magnetic field using a LabVIEW interface. The inputs from LabVIEW are executed by the NI DAQ device which generates sinusoidal signals to the power supplies for the electromagnetic coils. The camera provides real time visual feedback and records videos at 90 frames per second. The schematic for the control system is shown in Figure 2.

The electromagnetic coils are arranged in a configuration similar to Helmholtz coils. The distance between each pair of coils is the same as the outer diameter of the coils plus the thickness of the coils; whereas a true Helmholtz configuration has a distance equal to the radius of the coils. This configuration allows for the generation of a near-uniform magnetic field at the center of the coils. This type of magnetic field is necessary in order to apply a magnetic torque on the microrobots without introducing translational magnetic force. In other words, the magnetic field will cause the microrobots to align with the field without pulling the microrobots to move along the field gradient. This is crucial for this study because the uniform field ensures that the movements of the microrobots were caused by the oscillation of their bodies. The intensity of the magnetic field generated from a pair of coils can be calculated using a modified version of the Biot-Savart law [55]

(1) $$B_{\mathrm{coils}}=\frac{\mu_{0}nIR^{2}}{2{\left(R^{2}+x^{2}-2dx+d^{2}\right)}^{\frac{3}{2}}}+\frac{\mu_{0}nIR^{2}}{2{\left(R^{2}+x^{2}+2dx+d^{2}\right)}^{\frac{3}{2}}}$$

where µ_0_ is the permeability, *n* is the number of turns of wires per coil, *I* is the electrical current passing through the wires, *R* is the effective radius of the coil, *d* is the distance between the center of a coil pair, and *x* is the coil distance to a point. The modification from the original Biot-Savart law accounts for the distance between the pairs of coils for this particular configuration.

The magnetic field strength (mT), direction of the oscillating field, and oscillation frequency (Hz) of the field generated by the coils can be controlled through the control system. To create an oscillating magnetic field, two pairs of electromagnetic coils are needed. The resultant field ***B*** can be expressed as

(2) $$B=B_{0}\left[\begin{array}{c} -\left(1-\sin\left(c\pi \right)\right)\cos\left(\theta \right)\left(\left|\cos\left(\omega t\right)\right|+\frac{\sin\left(c\pi \right)}{\left(1-\sin\left(c\pi \right)\right)}\right)+\cos\left(c\pi \right)\sin\left(\theta \right)\sin\left(\omega t\right)\\ (1-\sin\left(c\pi \right))\sin\left(\theta \right)\left(\left|\cos\left(\omega t\right)\right|+\frac{\sin\left(c\pi \right)}{\left(1-\sin\left(c\pi \right)\right)}\right)+\cos\left(c\pi \right)\cos\left(\theta \right)\sin\left(\omega t\right) \end{array}\right]$$

where *B*_0_ is the amplitude of the magnetic field, ω is the oscillating frequency of the field, θ is the direction of oscillation, *c* is a coefficient that determines the angle of oscillation α, and t is time. The coefficient *c* and the angle of oscillation α will be discussed further in the proceeding section. In motion control experiments, the angle θ controls the movement direction and ω controls the movement speed. The oscillating magnetic fields generated using Equation (2) offer a few advantages over methods used for previous swimmers [36,39,40]: (1) they allow for swimming at any directions based on the angle θ, (2) they generate near uniform fields similar to Helmholtz coils, and (3) they maintain a near constant field strength for the full cycle of each oscillation.

### 2.3. Oscillatory Swimming Motion

**Figure 3:**
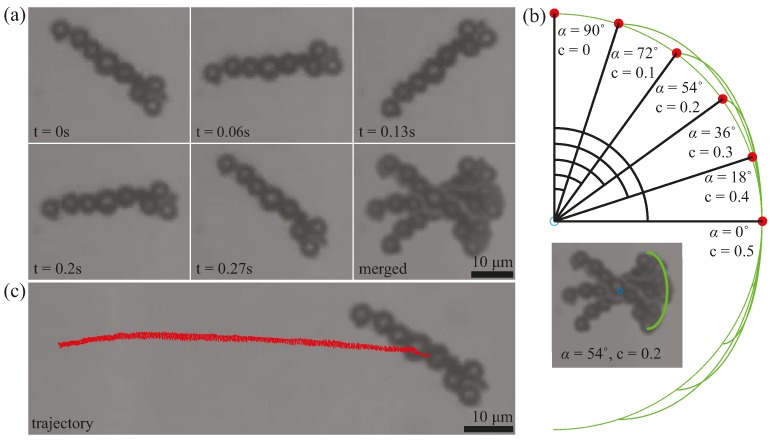
Oscillatory motion of the microrobots and magnetic field. (**a**) Frame by frame representation of a full oscillation cycle from *t* = 0 s to *t* = 27 s. (**b**) An illustration of six angles of oscillation α and the corresponding *c* values. The inset image shows the microrobot from (a) with an overlay arc at an angle of oscillation α of 54° and a corresponding *c* value of 0.2. (**c**) Translational motion of the oscillating microrobot in (a); also see Video S1. The scale bars are 10 µm.

The microrobots can undergo oscillating motion at any desired frequency using the parameter ω from Equation (2). A frame by frame representation of the oscillating motion of a microrobot is shown in Figure 3a. The microrobot oscillated at 4 Hz for one full cycle from *t* = 0 s to *t* = 0.27 s. The merged image shows the full range of the oscillation with an angle of oscillation (α) of 54°. Using the control parameter *c* in Equation (2), we can manipulate the angle of oscillation of the microrobots. In Figure 3b, six arcs are drawn using Equation (2) to represent full cycles of oscillation of the magnetic field at six angles of oscillation. The value of *B*_0_ does not matter here since *B*_0_ only serves as a scaling parameter. The frequency of oscillation ω also does not matter for the static representation of the oscillation. From the arcs, six different angles of oscillation are presented along with the corresponding *c* values. For example, the angle of oscillation of 54° from Figure 3a was obtained using *c* = 0.2 in Equation (2). It should be noted that *c* is a dimensionless coefficient used to yield a specific angle of oscillation α; the value of *c* does not have a physical meaning.

## 3. Results

### 3.1. Velocity Profile

To demonstrate the motion control capability, we characterized the velocities of the microrobots at various oscillating frequencies (0 Hz, 10 Hz, 20 Hz, and 30 Hz). For consistency, the value of *c* was kept at 0.2, which corresponded to an angle of oscillation of 54°. The magnetic field strength *B*_0_ was also kept at a constant value of 6.81 mT. The applied field was strong enough to oscillate the microrobots at the desired frequency and weak enough to keep the electromagnetic coils from overheating. For experiments, we placed the microrobots into a simple Polydimethylsiloxane (PDMS) chamber filled with water. The chamber was sealed as shown in Figure 2. The chamber was then placed inside the electromagnetic coil system.

A MATLAB tracking algorithm was used to analyze the motion of the microrobots. The algorithm performed image processing on videos that were recorded at 30 frames per second at 640 by 512 resolution. The image processing extracted the centroid position (*x*,*y*) of the microrobots at each consecutive frame. The process followed four steps: image binarization using a grayscale thresholding, structure definition based on pixel continuity, size thresholding to get rid of objects with outlier sizes, and calculation of the centroid. The centroids from each consecutive frame were used to calculate their velocities.

**Figure 4:**
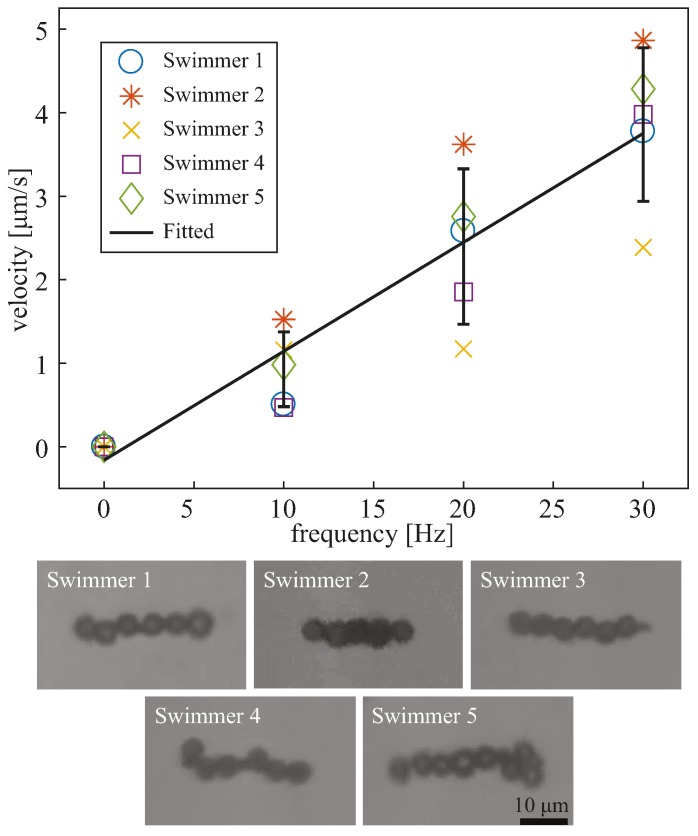
Velocity profiles for oscillating microrobots plotted against oscillation frequency. The plot includes five different microrobots. The fitted line is a linear fit of the mean velocities of the five microrobots. The error bars represent standard errors. The images below the plot show the geometry for each microrobot represented in the plot. The sharpness and contrast of the images were enhanced for visibility.

The velocity profiles and images of the five oscillating microrobots are shown in Figure 4. The five different microrobots were tracked and exhibited linear relationships between the swimming velocity and the osciliation frequency. The approxiate length of the microrobots were 29.11 µm, 23.63 µm, 28.06 µm, 27.30 µm, and 33.21 µm, respectively. The velocities were offset by subtracting the background flow. The background flow velocities were determined by observing the movement of the nonmotile microrobots at 0 Hz. After the offset, velocities at 0 Hz became 0 µm/s, and the velocities at other frequencies were offset accordingly by the background flow. It should be noted that the background velocities were very small due to how close the microrobots were to the surface. From these data, it can be seen that the velocities of the microrobots vary from one another due to their geometry. Due to the fabrication process, the geometry of the microrobots is not the same, therefore, we chose microrobots with lengths within 10 µm from each other. Although we measured the length of the microrobots, the images in Figure 4 show that geometrical variation between the microrobots goes beyond length, since some microbots are more linear than others; thus, we did not attempt make a conclusion based on length and speed. To draw a relationship between the geometry and swimming speed, precise geometry and deformation were needed, in order to examine the friction parallel and perpendicular to the bodies of the microrobots.

We applied a linear fit to the average velocities of the five robots with a slope of 0.13 and a *R*^2^ value of 0.99. The standard deviation for the error bars at 10, 20, and 30 Hz were 0.4478 µm/s, 0.9301 µm/s, and 0.9198 µm/s, respectively. The coefficient of variations were 0.4824, 0.3880, and 0.2384, respectively. The information for the fitted line in Figure 4 is listed in Table 1.

### 3.2. Motion Control

**Figure 5:**
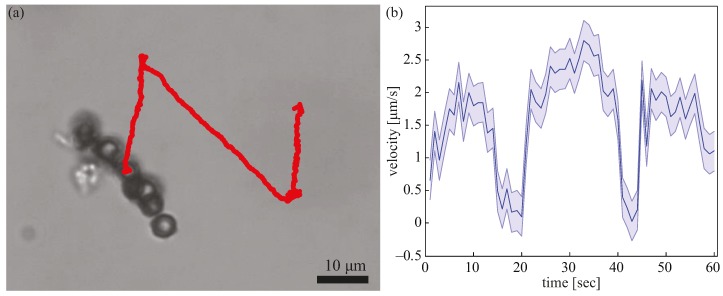
Steering of the microrobots. (**a**) An oscillating microrobot traveled in an *N* pattern. (**b**) The velocity profile plotted against time for the microrobot in (a).

Using Equation (2), the movement direction of the microrobots can be manipulated by changing the angle θ. To demonstrate the turning capability of the oscillating microrobots, a representative example of a microrobot that steered to travel in an *N* pattern is shown in Figure 5a. The oscillating frequency was kept at 15 Hz for the entire duration of the experiment. This illustrates the ability of making sharp turns while using the oscillating mode. The velocity of the microrobot was measures at one second intervals and is plotted in Figure 5b. The drops in velocity at around *t* = 15 s to *t* = 20 s, *t* = 40 s, and *t* = 43 s were attributed to pauses that were used to facilitate turning.

## 4. Discussion

The results of this work demonstrate movement speed control and directional control of magnetically actuated oscillating microrobots. The velocity profile in Figure 4 illustrated a linear relationship between movement speed and oscillation frequency. The *R*^2^ value of 0.99 of the linear curve fit suggests a high linear correlation between the velocity of the microrobots and the applied oscillation frequency. Furthermore, the relatively small coefficient of variance illustrates the consistencies between the velocity profiles of the microrobots. This linear relationship will be useful in controlling and predicting the movement speed by using the oscillation frequency as a control parameter. This will be particularly useful for developing a qualitative kinematic model that can be used for autonomous feedback control. Furthermore, the demonstration of sharp turns suggests that the microrobots can be controlled as omnidirectional robots, which is a common property among microrobots controlled using externally applied magnetic fields.

## 5. Conclusions

In summary, the demonstration of using oscillation as a mode of motion for particle based microrobots presents the possibility to move on or near surfaces where rotational motion might not be preferred. As experiments have shown, the microrobots can be assembled en masse using chemical and magnetic bonding. To show the viability of using oscillation as a strategy for locomotion, experiments were performed to characterize the velocity profiles of five different microrobots at incremental frequencies. The linearity of the velocity profiles demonstrated the feasibility of manipulating the movement speed by controlling the oscillation frequency. The steerability of the microrobot was also investigated, to demonstrate that both speed and direction control can be achieved using magnetically induced oscillation.

## Figures and Tables

**Table 1:** Values for the fitted line in Figure 4.

Frequency	Mean Velocity (µm/s)	Standard Deviation (µm/s)	Coefficient of Variations
10	0.9284	0.4478	0.4824
20	2.3973	0.9301	0.3880
30	3.8580	0.9198	0.2384

## Supplementary Materials

The following are available online at www.mdpi.com/2072-666X/8/2/46/s1. Video S1: On surface translational motion of an oscillating microrobot.
